# Phoenixin: More than Reproductive Peptide

**DOI:** 10.3390/ijms21218378

**Published:** 2020-11-08

**Authors:** Maria Billert, Agnieszka Rak, Krzysztof W. Nowak, Marek Skrzypski

**Affiliations:** 1 Department of Animal Physiology, Biochemistry and Biostructure, Poznań University of Life Sciences, 60-637 Poznań, Poland; maria.billert@up.poznan.pl (M.B.); kwnowak@up.poznan.pl (K.W.N.); 2 Department of Physiology and Toxicology of Reproduction, Institute of Zoology and Biomedical Research, Jagiellonian University in Kraków, 30-387 Kraków, Poland; agnieszka.rak@uj.edu.pl

**Keywords:** adipocytes, c4orf52, food intake, GPR173, metabolism, phoenixin, reproduction, smim20, thirst

## Abstract

Phoenixin (PNX) neuropeptide is a cleaved product of the Smim20 protein. Its most common isoforms are the 14- and 20-amino acid peptides. The biological functions of PNX are mediated via the activation of the GPR173 receptor. PNX plays an important role in the central nervous system (CNS) and in the female reproductive system where it potentiates LH secretion and controls the estrus cycle. Moreover, it stimulates oocyte maturation and increases the number of ovulated oocytes. Nevertheless, PNX not only regulates the reproduction system but also exerts anxiolytic, anti-inflammatory, and cell-protective effects. Furthermore, it is involved in behavior, food intake, sensory perception, memory, and energy metabolism. Outside the CNS, PNX exerts its effects on the heart, ovaries, adipose tissue, and pancreatic islets. This review presents all the currently available studies demonstrating the pleiotropic effects of PNX.

## 1. Introduction

The diverse biological effects of neuropeptides are of wide interest to researchers. Different identification strategies allow the discovery of novel peptides, including identification from biological activities, receptor or genomic approaches [1]. Based on bioinformatic analyses of evolutionary conserved sequences peptides, Samson et al., in 2008 discovered neuronostatin [2] and five years later, the same team identified phoenixin (PNX) [3]. Although it has not yet been ten years since then, many studies have shown that PNX exerts a variety of biological effects. It is worth noting that initial PNX studies focused on its role in the reproduction system [3,4], however no less important effects were observed, e.g., in memory and anxiety [5,6], as well as in glucose and lipid metabolism [7,8]. Some of these bioactivities exhibit features similar to other neuropeptides like orexins [9], ghrelin [10], or kisspeptin [11]. Moreover, in the hypothalamus, PNX is co-expressed with nesfatin-1 [12]. It could not be excluded that PNX effects are dependent on its interaction with other neuropeptides. Considering the broad spectrum of PNX activities and its expanding research area, in this review, we describe all currently known PNX biological effects.

## 2. Characterisation of Phoenixin and GPR173 Receptor

**Figure 1:**
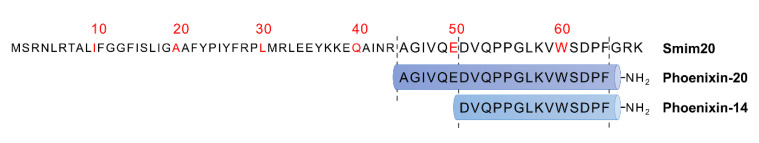
Amino acid sequence of PNX precursor peptide—Smim20 and two main PNX isoforms: PNX-20 and PNX-14.

Phoenixin neuropeptide was identified in 2013 by Yosten et al. [3] It was discovered by a bioinformatic algorithm based on the Human Genome Report data used for predicting unidentified and highly conserved peptide sequences. PNX is cleaved from the C-terminal small integral membrane protein 20 (Smim20), also known as C4orf52 [13]. Smim20 is a component of the mitochondrial translation regulation assembly intermediate of the cytochrome c oxidase complex, involved in the biogenesis of cytochrome c oxidase, and stabilizes the COX1 subunit [14]. The most common isoforms of PNX are amidated peptides composed of 14 and 20 amino acids (Figure 1) [3]. However, 17-, 26-, 36-, and 42-amino acid isoforms of PNX have also been predicted [3,15]. Phoenixin-14 amide (PNX-14) and phoenixin-20 amide (PNX-20) exhibit similar biological activities, whereas the nonamidated PNX is inactive [3]. The amino acid sequence of PNX is closely species-conserved and is identical in humans, rats, mice, bovines, and pigs. Only one amino acid differs in PNX-20 between humans and rodents [3]. The high degree of the conservation of peptide sequences across species indicates that PNX might be evolutionarily significant. During evolution, genomes of all organisms undergo mutations that change the amino acid sequences and in turn the peptide functions. In general, the resulting two organisms will be evolutionarily distant from each other, with an increased occurrence of amino acid substitutions. A small percentage of differences in the identity of PNX between different species may indicate a significant function of the peptide that is slightly mutated. A neuropeptide that undergoes minor interspecies mutations is neuropeptide Y [16].

The highest expression of PNX in rats was detected in the hypothalamus [3]. PNX is present in the paraventricular nucleus (PVN), supraoptic nucleus (SON), zona incerta, arcuate nucleus (Arc), dorsal hypothalamus, and ventromedial hypothalamus (VMH). In addition, this peptide was observed in the median eminence (ME) and the posterior as well as the anterior pituitary gland [3]. The neurosecretory hypothalamus magnocellular neurons secrete the PNX peptide into the blood [12]. On the other hand, the PNX present in the circulatory system may be secreted by other tissues. For example, both adipocytes and pancreatic islets of rat have been shown to secrete PNX [7,8].

PNX was found to be highly expressed in the spinal cord [3,13]. The peptide was also detected in the peripheral tissues such as the heart [3,17], thymus, esophagus, stomach [3], duodenum, jejunum, colon [3,18], pancreatic islets [8,18], adipose tissue [7,19], ovaries [19,20], and epidermis and dermis of the skin [15]. The spectrometric mass technique identified the differences in the expression between the PNX-14 and PNX-20 isoforms. Although the brain is dominated by the PNX-20 peptide, the more ubiquitous isoform expressed in the spinal cord and heart is PNX-14 [3,13].

A list of areas and tissues where PNX is expressed is provided in Table 1.

A deductive ligand–receptor matching strategy identified G protein-coupled receptor 173 (GPR173) as a putative receptor of PNX. Stein et al., demonstrated that siRNA-downregulated *GPR173* mRNA expression attenuated PNX-stimulated LH secretion induced by GnRH in female rats [4]. Consistently, the PNX activation of GPR173 was confirmed in the pituitary cells [4], GnRH- and kisspeptin-positive neurons [29], granulosa cells [20], and murine microglial BV2 cells [30]. GPR173 belongs to the Super Conserved Receptor Expressed in the Brain (SREB) family and is also termed as SREB3. The SREB family consists of three members: GPR27 (SREB1) and GPR85 (SREB2) in addition to GPR173. However, it was predicted that PNX interacts not only with GPR173 but also with other orphan receptors such as GPR15 and GPR25 [29]. Similar to PNX, GPR173 is predominantly expressed in the brain and in the gonadal areas [31].

In summary, PNX is a neuropeptide that is expressed and secreted not only in the CNS but also in the peripheral tissues. Its main isoforms are 14- and 20-amino acid peptides, which exhibit similar biological activity, and their expression is tissue-dependent. GPR173 is identified as a putative PNX receptor; however, the selective binding of PNX to this receptor has yet to be confirmed.

## 3. Phoenixin in Reproduction System

In the pioneering study that identified PNX, it was termed as a novel reproductive peptide [3]. PNX was found to be expressed in the anterior pituitary gland; however, the same study showed that PNX-20 could not modulate the secretion of LH from the pituitary cultures of female rats in vitro. It was observed that PNX-20 potentiated GnRH-stimulated LH secretion from those cultures. Moreover, in the pituitary gland, PNX increased the expression of the GnRH receptor and potentiated the GnRH receptor expression induced by GnRH. On the other hand, Stein et al., demonstrated that intracerebroventricular (icv) injection of PNX-20 simulated LH release in rats [4]. In the same in vivo model in male rats, PNX not only induced LH secretion but also increased the serum concentrations of FSH and testosterone without causing any changes in the level of GnRH [32]. Overall, these studies showed that the effects of PNX on the pituitary gland differed in vitro and in vivo, but their results collectively suggested that this peptide may be involved in controlling gonadotropin secretion.

Consistently, there is evidence indicating that PNX may play a role in controlling the estrous cycle in females. Yosten et al., showed that in Sprague-Dawley female rats, siRNA-mediated downregulation of PNX expression in the pituitary gland delayed the next estrous cycle by 2.3 days, which was associated with a prolonged diestrus 2 stage [3]. Furthermore, the downregulation of GPR173 mRNA expression in the pituitary gland was shown to be accompanied by a prolonged diestrus phase [4].

There is evidence that PNX affects not only the pituitary gland but also hypothalamic neurons. In the mHypoA-GnRH/GFP cell line representing GnRH population cells, PNX increases *GnRH* and GnRH receptor (*GnRHR*) mRNA expression, as well as GnRH secretion [29]. Treen et al., show that these findings are dependent on the cAMP/PKA pathway and are involved with regulation of transcription factors cAMP response element binding protein (CREB), *C/ebp-β*, and *Oct-1* [29].

The effects of PNX on the hypothalamus and pituitary and its modulatory effects on the secretion of LH and GnRH indicate that it is involved in the hypothalamic–pituitary–gonadal axis. PNX is expressed in the ovary [19] and ovarian follicles [20]. Moreover, the expression of *Smim20* increases during maturation. It was found that PNX-14 simulates the proliferation of human granulosa HGrC1 cells and maturation of ovarian follicles, and increases the number of ovulated oocytes [20]. In addition, PNX stimulates the expression of follicle development-related genes including *FshR*, *LhR*, and *Kitl* in human granulosa cells. The peptide also enhances estradiol production in granulosa cells by activating the cAMP/PKA pathway and stimulating the phosphorylation of CREB [20].

There is evidence that the effects of PNX on the secretion of reproductive hormones are mediated by its interaction with other peptides involved in reproduction. A study showed that nesfatin-1 may potentiate the effects of PNX-14 on the release of reproductive hormones such as LH, FSH, and testosterone in male rats [32]. Of note, nesfatin-1 is an anorexic neuropeptide involved in energy metabolism and glucose homeostasis [33]. PNX is highly co-expressed with nesfatin-1 in the hypothalamus [12]. Furthermore, icv injection of PNX-14 activates nesfatin-1 immunoreactive neurons in the brain [34]. Although nesfatin-1 is involved in reproduction [35], its interaction with PNX in the reproductive system is not clear. PNX-20 also modulates the hypothalamic neuropeptide, kisspeptin. In the mHypoA-Kiss/GFP-3 cell line, PNX increased the expression of *Kiss1* mRNA by cAMP/protein kinase A pathway and stimulated the phosphorylation of CREB [29]. It is worth noting that the contribution of PNX to controlling reproduction was also confirmed in fishes. It was found that in spotted scat (*Scatophagus argus*) PNX-14 stimulates expression of *GnRHR*, *Lh* and *Fsh* [36].

Several studies investigated the blood PNX levels in the reproductive system diseases. Increased PNX levels were observed in women who suffered from polycystic ovary syndrome (PCOS) [37]. This disease is characterized by elevated levels of LH and progesterone [38,39]. Furthermore, an increased nesfatin-1 level is also observed in PCOS [37]. In PCOS patients, a higher concentration of LH positively correlates with the PNX level [19,37]. Moreover, positive correlations have been observed for PNX with other reproductive hormones, including testosterone and progesterone [37]. Consistently, increased expression of the PNX precursor, *Smim20*, was observed in the ovaries, particularly in the periovarian adipose tissue, of PCOS female rats compared to healthy female rats. The ovaries of PCOS females displayed an increased production of PNX, which was associated with increased phosphorylation of ERK1/2 as well as PKA and Akt [19]. Nevertheless, it is rather unknown whether increased PNX levels in the circulation contributes to the development of PCOS or vice versa.

In summary, PNX plays a significant role in the reproductive system. In females, PNX stimulates LH secretion and modulates the duration of the diestrus stage. In addition, in the ovaries, it simulates the maturation of the ovarian follicles and increases the number of ovulated oocytes. Increased PNX levels are observed in PCOS females, which correlates with elevated levels of LH and progesterone.

## 4. Phoenixin in the Regulation of Food Intake and Thirst

PNX peptide was detected in the brain areas involved in controlling appetite such as the Arc, PVN, VMH, and the nucleus of the solitary tract (NTS). Therefore, several studies investigated the role of PNX in controlling food intake. Shalla et al., found that icv administration of PNX-14 during the light phase stimulated food intake but decreased intermeal intervals in adult male Sprague-Dawley rats [40]. The study also reported that PNX-14 increased the eating rate as well as the meal duration, but reduced intake during the dark phase. In addition, the authors of the study aimed to evaluate the effect of intraperitoneal (ip) injection on feeding behavior. However, intravenous PNX-14 administration was not found to have any effect on food intake measured during the light as well as the dark phase. Stimulation of food intake by PNX in rats was additionally confirmed by an independent study. Friedrich et al., reported that icv administration of PNX-14 stimulated food intake in rats [34]. A more detailed study was conducted on the SON and PVN and the medial part of the NTS. PNX administration was accompanied by an increased amount of immune-reactive c-Fos positive cells, c-Fos/nesfatin-1, and NUCB2/nesfatin-1 cells. Of note, in contrast to PNX-14, nesfatin-1 suppressed food intake [41]. Therefore, it was postulated that activation of nesfatin-1-positive neurons may be caused by gastric distension induced by increased food intake and/or contribute to meal termination [34]. Importantly, the orexigenic action of PNX described in rodents was also confirmed in fishes. In spotted scat, fasting stimulated *Smim20* mRNA expression while it was reduced after refeeding in the hypothalamus [21]. Therefore, the authors of the work speculated that in spotted scat, PNX may be an orexigenic factor [21]. Nevertheless, a recent study found a complex role for PNX in the regulation of food intake. Rajeswari et al., reported that in zebrafish, fasting (7 days) suppressed *Smim20* mRNA expression in the brain [42]. Moreover, the same study showed that ip injection of PNX-20 suppressed food intake [42]. Interestingly, inhibition of food intake was accompanied by an increased expression of *Cart* mRNA in the hypothalamus and suppressed the expression of ghrelin in the gut and hypothalamus [42]. However, as suggested by the authors, it cannot be excluded that the observed downregulation of food intake resulted from the different type of PNX administration (ip) [42]. There is limited knowledge on the role of PNX in controlling appetite in humans. However, it is worth noting that the blood PNX levels were decreased in malnourished anorectic patients and increased during body weight normalization [43]. Considering other orexigenic peptides such as ghrelin [44] and neuropeptide Y [45] that are elevated in patients who suffer from anorexia nervosa, this observation was surprising. Nevertheless, since PNX is produced in fat cells [7], lower PNX levels in anorexia nervosa may result from a reduced content in adipose tissue [43]. Furthermore, as pointed out by the authors of this work, the central level of PNX in anorexic patients remains unknown [43]. When discussing the role of PNX in reproduction, it is worth mentioning that there is evidence indicating that expression of *Smim20* and PNX putative receptor (*GPR173*) mRNA is modulated by nutritional and chemical factors. For example, it was found that *Smim20* mRNA was upregulated by palmitate, DHA and oleate and in murine immortalized hypothalamic neurons [46]. The authors of this work suggested that stimulation of PNX production by nutritional factors such as fatty acids may be a signal to promote reproductive processes. The mechanism by which these factors alter *Smim20* expression is not clear, but there is evidence that this process is not regulated by cAMP, NO, PKC or neuroinflammation [46]. On the other hand, expression of *Smim20* mRNA expression is downregulated by bisphenol A. However, this effect was observed in male hypothalamic cell lines, only. By contrast, bisphenol A promoted *Smim20* mRNA expression in female hypothalamic cell line. In addition, *Smim20* mRNA expression is also downregulated by bisphenol A in vivo in female Wistar rats [47]. Sex- and cell-depended effects of bisphenol A on *Smim20* mRNA expression remains unknown. It was also found that GPR173 expression in murine hypothalamic cell lines is downregulated by palmitate or bisphenol A via a p38-dependent manner [22]. However, more research is needed to elucidate the physiological relevance of this finding.

In addition to the modulation of food intake, PNX is implicated in thirst regulation. The study conducted by Shalla et al. [40] showed that in the light phase, PNX-20 increased water intake, but the difference was not statistically significant (*p* = 0.105). However, stimulation of water intake by PNX was documented by a recent study. It was found that PNX-20 administration stimulated water intake in both male and female rats [25]. Interestingly, PNX stimulation of water intake was attenuated by the angiotensin receptor blocker [25]. Nevertheless, the molecular mechanism of this effect is unclear. Importantly, GPR173 receptors, which are putative receptors of PNX, are differentially expressed during the estrous cycle, which may suggest a potential role for PNX in fluid retention during different physiological conditions [25].

In summary, rodent studies collectively showed that centrally administrated PNX stimulates food intake. On the other hand, PNX was found to suppress appetite in zebrafish [42]. Thus, more research including human studies is needed to elucidate the role of PNX in the regulation of food intake. Moreover, rodent studies clearly proved that PNX promotes water intake.

## 5. Phoenixin in Memory and Anxiety

Based on the fact that PNX regulates GnRH production in the CNS and that GnRH is involved in learning and memory, the role of PNX in these processes was explored. Jiang et al. demonstrated that icv injection of PNX-14 prolonged the retention of object memory and facilitated object recognition memory in mice [5]. In addition, memory-enhancing effects were observed when PNX-14 was injected into the hippocampus. However, these effects were reduced when a GnRHR antagonist (Cetrorelix) was used [5]. Memory impairment and cognitive disturbances accompany Alzheimer’s disease (AD). One of the major hypotheses regarding the causes of AD is the accumulation of amyloid β (Aβ) in the brain [48]. In addition to scopolamine-induced cognitive dysfunctions, as well as memory impairment induced by icv injection of Aβ in mice, PNX-14 significantly ameliorated the memory deficiency and location recognition memory [5]. In addition, it was found that plasma PNX levels did not correlate with any of the cognitive and metabolic parameters in AD patients. In mild cognitive impairment, plasma PNX concentration negatively correlates with logical memory, whereas it positively correlates with metabolic parameters including body mass index (BMI), systolic blood pressure, and high-density lipoprotein level [49]. However, it should be noted that gender differences were not taken into consideration in the study.

The role of PNX in behavior has also been studied in the context of anxiety. In behavioral tests used to evaluate anxiety disorder-related behaviors in rodents, elevated plus maze test, and open-field test, icv injection of PNX-14 dose-dependently increased the anxiolytic effects in mice [6]. Moreover, not only PNX-14 but also icv injection of PNX-20 induces anxiolytic effects, which may prove the significant role of PNX as an antianxiety agent [6]. However, Yuruye et al., did not observe any differences in the open-field test study in mice treated with PNX-14 [49]. Interestingly, the anxiolytic effects of PNX in mice are dependent on the GnRHR. Jiang et al., reported that Cetrorelix attenuated these effects [6]. Consistently, in a human study, Hofmann et al., showed that plasma PNX levels were negatively correlated with anxiety scores in obese men [50]. However, considering that PNX-20 simulates vasopressin but not oxytocin secretion, the antianxiety effects of PNX appear surprising. Both hormones are involved in anxiety and social behaviors but present opposite effects. Vasopressin stimulates anxiety- and depression-related behaviors, whereas oxytocin acts anxiolytically and as an anti-depressive agent [51]. Of note, kisspeptin-13 (KP-13), the neuropeptide stimulating GnRH secretion, displays increased spontaneous locomotor activity and potentiates anxiety in rats [52]. On the other hand, KP-13 enhances memory formation and extends memory retention induced by Aβ in mice, in a GPR54 (KP-13 receptor)- and GnRHR-dependent manner [53]. It is worth noting that although PNX is co-expressed with nesfatin-1 in the hypothalamus, it exerts an opposite effect in terms of anxiety. There is evidence showing that nesfatin-1 increases anxiety behavior and fear-related responses in rats [54]. Thus, the effect of PNX as an anxiolytic peptide is probably complex and requires more extensive research.

## 6. Other Effects of Phoenixin in Central Nervous System

In the spinal cord, PNX-14 is expressed in the dorsal root ganglion cells [13,15]. The immunoreactivity of PNX-14-positive cells in the superficial layer of the dorsal horn and in the skin in mice suggested the role of PNX in sensory processes. Subcutaneous injection of PNX-14 to the nape of the neck elicited repetitive scratching bouts in mice. Consistently, the injection of nalfurafine, an effective kappa opioid receptor agonist, attenuated chemically diverse pruritogens, 20 min before PNX-14 suppressed the scratching bouts induced by the peptide [15]. Of note, PNX-14, but not PNX-20, exerts this effect. Peptides such as substance P and calcitonin gene-related peptides play a similar role as substances that affect the pruritus [55]. In addition to an itching sensation, PNX is involved in nociception [13]. In a pain model using acetic acid in mice, intrathecal injection of PNX-14 reduced the number of writhes compared to vehicle animals. However, PNX showed no effect on tail-flick latency [13]. In conclusion, PNX is expressed in the dorsal horn of the spinal cord in mice and is involved in sensory processes. Subcutaneous injection of PNX-14 to the neck elicited excessive scratching in mice; however, if injected intrathecally, PNX reduced tail-flick latency in the visceral pain model using acetic acid.

PNX is highly expressed in the nucleus of the solitary tract (NST) [3]. This brain area is involved in the regulation of reproduction and is responsive to stress. A recent study has proved that PNX exerts effects on membrane potential as well as spike frequency of NST neurons [56]. Moreover, these effects can be dependent on the environment and induced by corticosterone treatment physiological stress. Considering that PNX is involved with reproduction and that stress factors can modulate the PNX effects on NST neurons, authors have suggested that PNX signaling may play an important role in the modulatory effects of stress in reproduction [56]. Furthermore, it is worth mentioning that restraint stress decreases plasma PNX levels in male rats, but in a less pronounced manner than changes in plasma cortisol levels [57]. Although the PNX contribution to the hypothalamic–pituitary–adrenal axis appears to be certain, its direct or indirect role is not clear and requires further research.

Immunodetection of PNX is observed in the SON and PVN of the hypothalamus, the areas controlling the homeostasis of fluid and electrolytes [3]. Hypothalamic magnocellular neurons transmit the axon through ME into the posterior pituitary gland where predominantly oxytocin and vasopressin are released [58]. In rats, PNX-20 simulates vasopressin but not oxytocin secretion from hypothalamic–neurohypophyseal explants. Moreover, it increases water intake in response to fluid deprivation [59].

It is evident that PNX is involved in regulating body core temperature in mice [6]. PNX-14 or PNX-20 injected into the lateral ventricles in conscious mice significantly decreased the animal’s core temperature through a GnRHR-dependent mechanism. Considering the co-expression of PNX with nesfatin-1 and PNX-induced expression of Kiss1, this observation is apparently surprising as icv injection of nesfatin-1 and KP-13 increases the body core temperature [52,60]. However, the PNX effect is dependent on the GnRHR, and Cetrorelix treatment in mice inhibited the hypothermic effect of PNX [6]. To the best of our knowledge, there is no evidence on the role of GPR173 in thermoregulation, and the role of PNX in the modulation of the body core temperature may be complex.

## 7. Phoenixin as Modulator of Lipid and Glucose Metabolism

The role of PNX in controlling metabolism and energy hemostasis is poorly described by the research data. Several studies found that the blood PNX level depends on body mass. PNX was shown to be positively correlated with BMI in women [37]. Considering that obesity results from fat tissue hypertrophy and hyperplasia [61], our group attempted to study the effects of PNX on white adipogenesis. The results showed that *GPR173* mRNA is expressed in rodent white preadipocytes as well as mature adipocytes [7]. Moreover, we found that PNX peptide is produced and secreted in mature white adipocytes [7] and that in vitro PNX-14 potentiates the proliferation of 3T3-L1 and rat white primary preadipocytes. Finally, we reported that PNX-14 promotes the differentiation of 3T3-L1 as well as rat primary preadipocytes into mature white fat cells. Stimulation of the differentiation of white fat precursor cells into mature adipocytes was found to be mediated via a cAMP-dependent mechanism [7]. In summary, these results indicate that PNX-14 may be involved in the formation of white fat tissue by promoting the proliferation, as well as the differentiation, of preadipocytes into adipocytes. Nevertheless, more studies are required to elucidate the role of PNX in the metabolism of fat tissue and endocrine activities.

Endocrine pancreas plays a pivotal role in controlling the metabolism of glucose and lipids. Pancreatic alpha and beta cells produce and release glucagon and insulin, which differentially modulate the homeostasis of lipids and glucose. Insulin suppresses postprandial glucose levels, promotes lipogenesis, and decreases lipolysis. By contrast, glucagon stimulates gluconeogenesis and promotes lipolysis during negative energy balance [62]. Abnormalities in the functions of alpha and beta cells are a hallmark of type 1 and type 2 diabetes mellitus [63,64,65]. There is evidence that the biology of these cells may be modulated by PNX. An initial study found that the PNX peptide is present in the pancreatic islets in rats [18]. PNX was detected in the periphery of pancreatic islets, which is composed of alpha cells; thus, in pancreatic islets, PNX may be produced by glucagon-positive cells [18]. However, using double immunofluorescence, we detected PNX peptides in both glucagon- and insulin-positive cells [8]. Moreover, we found that PNX was expressed in insulin-producing rat INS-1E cells. In addition, using freshly isolated rat pancreatic islets, we observed that PNX secretion is promoted by high glucose concentration [8]. Overall, these results suggested that PNX may be involved in modulating the functions of beta as well as alpha cells. Indeed, using INS-1E cells, we found that PNX-14 stimulated cell proliferation in an ERK1/2- and Akt-dependent manner [8]. In addition, PNX-14 promoted insulin mRNA expression in INS-1E cells. Based on these, we reported that PNX-14 potentiated glucose-induced insulin secretion in INS-1E cells and in isolated rat pancreatic islets via a cAMP/Epac-dependent mechanism [8]. On the other hand, the role played by PNX in pancreatic alpha cells remains to be studied. In summary, these data show that PNX may contribute to the modulation of energy homeostasis and metabolism by controlling the neogenesis and secretion of insulin. Nevertheless, more in vivo studies are required to confirm this speculation.

## 8. Cell-Protective and Anti-Inflammatory Effects of Phoenixin

There is evidence indicating that PNX-20 is implicated in controlling neuronal mitochondrial functions. A study on neuronal M17 cells showed that PNX-20 promoted the expression of *PGC-1α*, *NRF-1*, and *TFAM*, which suggested its role in mitochondrial biogenesis [66]. Consequently, the same study found that PNX-20 increased mitochondrial DNA content, mitochondrial gene expression, oxygen consumption rate, and intracellular ATP content [66]. However, the effects of PNX-20 on mitochondrial functions were not observed in cells in which GPR173 was downregulated [66]. Thus, the study showed that PNX in neurons may be involved in controlling mitochondrial biogenesis in a GPR173-dependent manner. In addition, PNX was observed to exhibit protective effects in astrocytes. Wang et al. reported that PNX-14 protects against LPS-induced cell damage and inflammation in mouse astrocytes [67]. Consistently, Zeng et al. found that PNX-20 attenuated LPS-induced inflammation in microglial cells [68]. The anti-inflammatory effects of PNX were mediated through the inhibition of TxNIP-mediated NLRP3 inflammasome activation [68]. In summary, PNX promotes mitochondrial biogenesis and protects cells from inflammation.

In addition, the role of PNX in ischemia/reperfusion (I/R) processes in the heart and in microglial cells of the brain was explored [17,30]. The current available studies on the heart show the highest expression of PNX in the peripheral tissues [3,13]. The peptide directly affects the myocardial cells, and reduces contractility and relaxation in a dose-dependent manner [17]. This effect of PNX involves an increase in the phosphorylation of Akt, eNOS, and ERK1/2. Concomitantly, PNX does not alter the coronary pressure and heart rate. Moreover, Rocca et al. showed that PNX administered at the reperfusion phase of I/R acted cardioprotectively, leading to smaller infarct size and a better systolic recovery in the isolated and Langendorff-perfused rat heart [17]. The cardioprotective effects were dependent on PNX-induced activation of RISK and SAFE cascades and inhibition of apoptosis. Considering that PNX is highly expressed in the heart, its exogenous administration does not exclude the para- or autocrine circuit.

On the other hand, increased plasma PNX level was observed in the postprandial phase in male rats fed a standard diet but not in animals on high-fat feed [17]. Because plasma PNX levels are augmented in obesity, probably local production of PNX could be inhibited in myocardial cells. As Rocca et al. have suggested, the inability of the heart to increase PNX secretion as a result of ischemia may increase the risk of heart injury in obese subjects [17].

The role of PNX in I/R and ischemic stroke was explored in microglia, and the effects of PNX on cell death and neuronal damage were studied [30]. Oxygen-glucose deprivation/reperfusion leads to acute inflammatory response and increases the secretion of proinflammatory cytokines and reactive oxygen species (ROS) in microglia [69]. Ma et al., have demonstrated that PNX protects against neuronal damage and inhibits cell death in murine microglial BV2 cells. The anti-inflammatory activity of PNX is associated with decreased expression of cytokines, including tumor necrosis factor-α, interleukin-1β, and interleukin-6. Moreover, PNX reduces the release of ROS and increases the production of anti-inflammatory glutathione [69]. Consistently, the protective effects of PNX against I/R injury reduces the infarct volume as well as suppressing the microglia activation in a middle cerebral artery occlusion rat model.

The ability of PNX to protect against oxygen-glucose deprivation/reoxygenation injury was also reported in human bEnd.3 brain endothelial cells [70]. It was found that in these cells upon oxygen-glucose deprivation/reoxygenation injury, PNX attenuates oxidative stress via suppression of ROS overproduction and downregulation of HMGB1 expression. Of note, HMGB1 was identified as a key mediator of immune response during ischemic stroke [71]. The same study found that PNX increases endothelial monolayer permeability via KLF2-dependet upregulation of occludin expression [70]. These data collectively indicated that PNX may improve blood–brain barrier function in patients who suffer from ischemic stroke.

A recently published animal study showed that PNX is able to protect against high-fat diet (HF)-induced non-alcoholic fatty liver disease in mice [72]. It was found that in mice with experimentally induced non-alcoholic fatty liver disease, PNX suppressed circulating levels of alanine and aspartate aminotransferases, cholesterol, and triglyceride as well as attenuating lipid deposition in the liver. The same study showed that beneficial effects of PNX administration on HFD-induced liver disease were mediated via attenuation of AMPK/SIRT activation [72].

In addition, a very recent study reported cell-protective effects of PNX and its receptor in isolated human dental pulp cells [73]. It was found that acting on these cells, PNX attenuates lipopolysaccharide-induced release of LDH as well as secretion of inflammatory mediators such as IL-6, MCP-1, VCAM-1, ICAM-1, MMP-2, and MMP-9. In addition, the same study proved that the anti-inflammatory effects of PNX on LPS-induced injury in dental pulp cells was mediated through attenuation of TL4 expression and suppression of NF-kB activation.

Considering the cardio- and cerebroprotective effects of PNX in I/R injury, it can be assumed that this peptide also protects and inhibits inflammatory processes in other tissues.

## 9. Concluding Remarks

**Figure 2:**
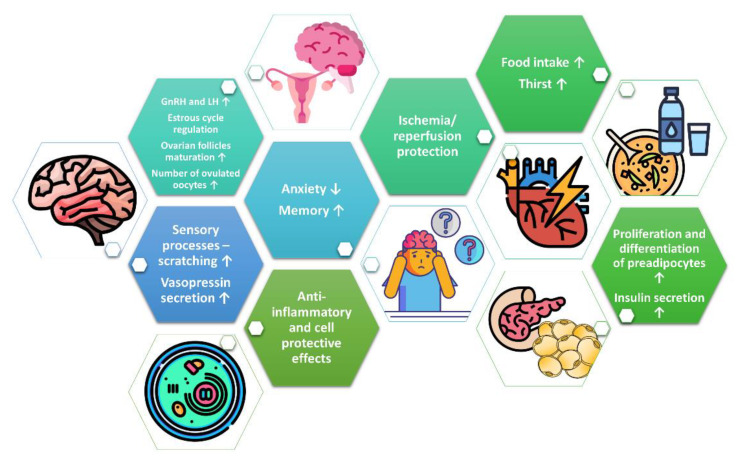
Summary of the biological effects of PNX.

In summary, PNX is implicated in reproduction, behavior, memory, sensory processes, fluid homeostasis, food intake, and glucose as well as in lipid metabolism (Figure 2). It is evident that PNX promotes the secretion of gonadotropins and steroid hormones. In addition, several studies have demonstrated that PNX displays cell-protective effects. Data indicate that the biological effects of PNX are mediated through the GPR173 receptor. Nevertheless, more studies are required to characterize the role of PNX and explain the therapeutic potential of PNX and its receptor(s) in human diseases. Although the existing research shows the pleiotropic effects of PNX, there is still a lack of studies confirming the role of PNX under in vivo conditions, especially in humans.

## Figures and Tables

**Table 1:** The semi-quantitative estimation of phoenixin-like immunoreactivity (PNX-li) and *Smim20* and *GPR173* mRNA expression in rat (R), mouse (M), human (H), pig (P), zebrafish (Zf), Scatophagus argus (Sa). −, no expression; +, low expression level; ++, medium expression level; +++, high expression level.

Area	PNX-li	*Smim20* mRNA	*GPR173* mRNA	Species	Publications
**Central nervous system**					
Hypothalamus (without nucleus division)	+++	+++	+++	Sa, R, Zf	[17,19,21,22,23,24]
Periventricular Nucleus	+++		+++	R	[3,4]
Paraventricular Nucleus	+++		++	R	[3,4,12]
Zona Incerta	++			R	[3]
Ventromedial Hypothalamus	++		+++	R	[3,4,12]
Supraoptic Nucleus	+++		+++	R	[3,4,18,25,26]
Lateral Hypothalamus	++		++	R	[3,4,12]
Substantia Nigra	++			R	[3]
Edinger–Westphal Nucleus	++			R	[3]
Nucleus Tractus Solitarius	++			R	[3,13,18]
Central Amygdaloid Nucleus	+++		+++	R	[4,18,26]
Arcuate Nucleus	+		++	R	[4,18,26]
Raphe Pallidus	+			R	[18,26]
Area Postrema	++			R	[18]
Median Eminence	++			R	[3]
Pituitary	++			Sa	[3,21]
Anterior Pituitary Lobe	+			R	[3]
Posterior Pituitary Lobe	+			R	[3]
Cerebrum	+			R	[3]
Pons	+			R	[3]
Spinal Cord	+++			R, M, P	[13,18,26,27]
Dorsal Root Ganglion	+++			R, M	[13,15]
**Peripheral tissues**					
Heart	+++	++		R, Sa, Zf	[3,13,17,21,23]
Thymus	++			R	[3]
Lung	++			R	[3]
Gill	+	+		Sa, Zf	[21,23]
Oesophagus	++			R	[3]
Stomach	++			R, Sa	[3,21]
Duodenum	++	++		R, Zf	[3,18,23]
Jejunum	+++			R	[3,18]
Ileum	++			R	[3,18]
Colon	−/+			R	[3,18]
Pancreas	++			R	[3,8,18]
Liver		++		Zf	[23]
Adipocytes	++	++	++	R, M	[7,19]
Kidney	++			R, Sa	[3,21]
Spleen	++			R, Sa	[3,21]
Ovary	++	++	++	H, Sa, R, Zf	[20,21,23,24]
Ovarian follicles	++	++	++	H, R	[19,20]
Testis	+	+		Sa, R, Zf	[17,21,23]
Muscle	+			R, Sa	[21,28]
Skin	++	++		M, Zf	[15,23]

## Abbreviations

AD	Alzheimer’s disease
Arc	Arcuate nucleus
Aβ	Amyloid β
COX1	Cytochrome C oxidase subunit 1
CREB	cAMP response element-binding protein
DHA	Dioxoheptanoic acid
ERK1/2	Extracellular signal-regulated kinases 1/2
FSH	Follicle-stimulating hormone
GnRH	Gonadotropin-releasing hormone
GnRHR	Gonadotropin-releasing hormone receptor
GPR173	G protein-coupled receptor 173
KP-13	Kisspeptin-13
LDH	Lactate dehydrogenase
LH	Luteinizing hormone
NTS	Nucleus of the solitary tract
NRF1	Nuclear respiratory factor 1
PCOS	Polycystic ovary syndrome
PGC-1α	Peroxisome proliferator-activated receptor gamma coactivator 1-alpha
PNX	Phoenixin
Smim20	Small integral membrane protein 20
SREB	Super Conserved Receptor Expressed in Brain
TFAM	Mitochondrial transcription factor A
VMH	Ventromedial nucleus of the hypothalamus

## References

[1] Corbiere A., Vaudry H., Chan P., Walet-Balieu M.L., Lecroq T., Lefebvre A., Pineau C., Vaudry D. (2019). Strategies for the Identification of Bioactive Neuropeptides in Vertebrates. Front. Neurosci..

[2] Samson W.K., Zhang J.V., Avsian-Kretchmer O., Cui K., Yosten G.L., Klein C., Lyu R.M., Wang Y.X., Chen X.Q., Yang J. (2008). Neuronostatin encoded by the somatostatin gene regulates neuronal, cardiovascular, and metabolic functions. J. Biol. Chem..

[3] Yosten G.L., Lyu R.M., Hsueh A.J., Avsian-Kretchmer O., Chang J.K., Tullock C.W., Dun S.L., Dun N., Samson W.K. (2013). A novel reproductive peptide, phoenixin. J. Neuroendocrinol..

[4] Stein L.M., Tullock C.W., Mathews S.K., Garcia-Galiano D., Elias C.F., Samson W.K., Yosten G.L. (2016). Hypothalamic action of phoenixin to control reproductive hormone secretion in females: Importance of the orphan G protein-coupled receptor Gpr173. Am. J. Physiol. Regul. Integr. Comp. Physiol..

[5] Jiang J.H., He Z., Peng Y.L., Jin W.D., Wang Z., Mu L.Y., Chang M., Wang R. (2015). Phoenixin-14 enhances memory and mitigates memory impairment induced by Abeta1-42 and scopolamine in mice. Brain Res..

[6] Jiang J.H., He Z., Peng Y.L., Jin W.D., Mu J., Xue H.X., Wang Z., Chang M., Wang R. (2015). Effects of Phoenixin-14 on anxiolytic-like behavior in mice. Behav. Brain Res..

[7] Billert M., Wojciechowicz T., Jasaszwili M., Szczepankiewicz D., Wasko J., Kazmierczak S., Strowski M.Z., Nowak K.W., Skrzypski M. (2018). Phoenixin-14 stimulates differentiation of 3T3-L1 preadipocytes via cAMP/Epac-dependent mechanism. Biochim. Biophys. Acta Mol. Cell Biol. Lipids.

[8] Billert M., Kolodziejski P.A., Strowski M.Z., Nowak K.W., Skrzypski M. (2019). Phoenixin-14 stimulates proliferation and insulin secretion in insulin producing INS-1E cells. Biochim. Biophys. Acta Mol. Cell Res..

[9] Soya S., Sakurai T. (2020). Evolution of Orexin Neuropeptide System: Structure and Function. Front. Neurosci..

[10] Kim S., Nam Y., Shin S.J., Park Y.H., Jeon S.G., Kim J.I., Kim M.J., Moon M. (2020). The Potential Roles of Ghrelin in Metabolic Syndrome and Secondary Symptoms of Alzheimer’s Disease. Front. Neurosci..

[11] Navarro V.M. (2020). Metabolic regulation of kisspeptin—The link between energy balance and reproduction. Nat. Rev. Endocrinol..

[12] Palasz A., Rojczyk E., Bogus K., Worthington J.J., Wiaderkiewicz R. (2015). The novel neuropeptide phoenixin is highly co-expressed with nesfatin-1 in the rat hypothalamus, an immunohistochemical study. Neurosci. Lett..

[13] Lyu R.M., Huang X.F., Zhang Y., Dun S.L., Luo J.J., Chang J.K., Dun N.J. (2013). Phoenixin: A novel peptide in rodent sensory ganglia. Neuroscience.

[14] Dennerlein S., Oeljeklaus S., Jans D., Hellwig C., Bareth B., Jakobs S., Deckers M., Warscheid B., Rehling P. (2015). MITRAC7 Acts as a COX1-Specific Chaperone and Reveals a Checkpoint during Cytochrome c Oxidase Assembly. Cell Rep..

[15] Cowan A., Lyu R.M., Chen Y.H., Dun S.L., Chang J.K., Dun N.J. (2015). Phoenixin: A candidate pruritogen in the mouse. Neuroscience.

[16] Elphick M.R., Mirabeau O., Larhammar D. (2018). Evolution of neuropeptide signalling systems. J. Exp. Biol..

[17] Rocca C., Scavello F., Granieri M.C., Pasqua T., Amodio N., Imbrogno S., Gattuso A., Mazza R., Cerra M.C., Angelone T. (2018). Phoenixin-14: Detection and novel physiological implications in cardiac modulation and cardioprotection. Cell. Mol. Life Sci..

[18] Prinz P., Scharner S., Friedrich T., Schalla M., Goebel-Stengel M., Rose M., Stengel A. (2017). Central and peripheral expression sites of phoenixin-14 immunoreactivity in rats. Biochem. Biophys. Res. Commun..

[19] Kalamon N., Blaszczyk K., Szlaga A., Billert M., Skrzypski M., Pawlicki P., Gorowska-Wojtowicz E., Kotula-Balak M., Blasiak A., Rak A. (2020). Levels of the neuropeptide phoenixin-14 and its receptor GRP173 in the hypothalamus, ovary and periovarian adipose tissue in rat model of polycystic ovary syndrome. Biochem. Biophys. Res. Commun..

[20] Nguyen X.P., Nakamura T., Osuka S., Bayasula B., Nakanishi N., Kasahara Y., Muraoka A., Hayashi S., Nagai T., Murase T. (2019). Effect of the neuropeptide phoenixin and its receptor GPR173 during folliculogenesis. Reproduction.

[21] Wang M., Deng S.P., Chen H.P., Jiang D.N., Tian C.X., Yang W., Wu T.L., Zhu C.H., Zhang Y., Li G.L. (2018). Phoenixin participated in regulation of food intake and growth in spotted scat, Scatophagus argus. Comp. Biochem. Physiol. B Biochem. Mol. Biol..

[22] McIlwraith E.K., Loganathan N., Belsham D.D. (2019). Regulation of Gpr173 expression, a putative phoenixin receptor, by saturated fatty acid palmitate and endocrine-disrupting chemical bisphenol A through a p38-mediated mechanism in immortalized hypothalamic neurons. Mol. Cell. Endocrinol..

[23] Rajeswari J.J., Unniappan S. (2020). Phoenixin-20 Stimulates mRNAs Encoding Hypothalamo-Pituitary-Gonadal Hormones, is Pro-Vitellogenic, and Promotes Oocyte Maturation in Zebrafish. Sci. Rep..

[24] Suszka-Switek A., Palasz A., Filipczyk L., Menezes I.C., Mordecka-Chamera K., Angelone T., Bogus K., Bacopoulou F., Worthington J.J., Wiaderkiewicz R. (2019). The GnRH analogues affect novel neuropeptide SMIM20/phoenixin and GPR173 receptor expressions in the female rat hypothalamic-pituitary-gonadal (HPG) axis. Clin. Exp. Pharmacol. Physiol..

[25] Haddock C.J., Almeida-Pereira G., Stein L.M., Yosten G.L.C., Samson W.K. (2020). A novel regulator of thirst behavior: Phoenixin. Am. J. Physiol. Regul. Integr. Comp. Physiol..

[26] Friedrich T., Schalla M.A., Lommel R., Goebel-Stengel M., Kobelt P., Rose M., Stengel A. (2020). Restraint stress increases the expression of phoenixin immunoreactivity in rat brain nuclei. Brain Res..

[27] Lepiarczyk E., Bossowska A., Majewska M., Skowronska A., Kaleczyc J., Majewski M. (2020). Distribution and chemical coding of phoenixin-immunoreactive nerve structures in the spinal cord of the pig. Ann. Anat..

[28] Nguyen T.V., Rotllant G.E., Cummins S.F., Elizur A., Ventura T. (2018). Insights Into Sexual Maturation and Reproduction in the Norway Lobster (Nephrops norvegicus) via in silico Prediction and Characterization of Neuropeptides and G Protein-coupled Receptors. Front. Endocrinol..

[29] Treen A.K., Luo V., Belsham D.D. (2016). Phoenixin Activates Immortalized GnRH and Kisspeptin Neurons Through the Novel Receptor GPR173. Mol. Endocrinol..

[30] Ma H., Su D., Wang Q., Chong Z., Zhu Q., He W., Wang W. (2020). Phoenixin 14 inhibits ischemia/reperfusion-induced cytotoxicity in microglia. Arch. Biochem. Biophys..

[31] Matsumoto M., Saito T., Takasaki J., Kamohara M., Sugimoto T., Kobayashi M., Tadokoro M., Matsumoto S., Ohishi T., Furuichi K. (2000). An evolutionarily conserved G-protein coupled receptor family, SREB, expressed in the central nervous system. Biochem. Biophys. Res. Commun..

[32] Guvenc G., Altinbas B., Kasikci E., Ozyurt E., Bas A., Udum D., Niaz N., Yalcin M. (2019). Contingent role of phoenixin and nesfatin-1 on secretions of the male reproductive hormones. Andrologia.

[33] Schalla M.A., Stengel A. (2018). Current Understanding of the Role of Nesfatin-1. J. Endocr. Soc..

[34] Friedrich T., Schalla M.A., Scharner S., Kuhne S.G., Goebel-Stengel M., Kobelt P., Rose M., Stengel A. (2019). Intracerebroventricular injection of phoenixin alters feeding behavior and activates nesfatin-1 immunoreactive neurons in rats. Brain Res..

[35] Kim J., Yang H. (2012). Nesfatin-1 as a new potent regulator in reproductive system. Dev. Reprod..

[36] Wang M., Chen H.P., Zhai Y., Jiang D.N., Liu J.Y., Tian C.X., Wu T.L., Zhu C.H., Deng S.P., Li G.L. (2019). Phoenixin: Expression at different ovarian development stages and effects on genes ralated to reproduction in spotted scat, Scatophagus argus. Comp. Biochem. Physiol. B Biochem. Mol. Biol..

[37] Ullah K., Ur Rahman T., Wu D.D., Lin X.H., Liu Y., Guo X.Y., Leung P.C.K., Zhang R.J., Huang H.F., Sheng J.Z. (2017). Phoenixin-14 concentrations are increased in association with luteinizing hormone and nesfatin-1 concentrations in women with polycystic ovary syndrome. Clin. Chim. Acta.

[38] Mortimer R.H., Lev-Gur M., Freeman R., Fleischer N. (1978). Pituitary response to bolus and continuous intravenous infusion of luteinizing hormone-releasing factor in normal women and women with polycystic ovarian syndrome. Am. J. Obstet. Gynecol..

[39] Jeffcoate S.L., Brooks R.V., London D.R., Prunty F.T., Rhodes P. (1968). Secretion of C19-steroids and oestrogens in the polycystic ovary syndrome. Ovarian studies in vivo and in vitro (including studies in vitro on a coincidental granulosa cell tumour). J. Endocrinol..

[40] Schalla M., Prinz P., Friedrich T., Scharner S., Kobelt P., Goebel-Stengel M., Rose M., Stengel A. (2017). Phoenixin-14 injected intracerebroventricularly but not intraperitoneally stimulates food intake in rats. Peptides.

[41] Stengel A., Goebel M., Tache Y. (2011). Nesfatin-1: A novel inhibitory regulator of food intake and body weight. Obes. Rev..

[42] Rajeswari J.J., Blanco A.M., Unniappan S. (2020). Phoenixin-20 suppresses food intake, modulates glucoregulatory enzymes, and enhances glycolysis in zebrafish. Am. J. Physiol. Regul. Integr. Comp. Physiol..

[43] Palasz A., Tyszkiewicz-Nwafor M., Suszka-Switek A., Bacopoulou F., Dmitrzak-Weglarz M., Dutkiewicz A., Slopien A., Janas-Kozik M., Wilczynski K.M., Filipczyk L. (2019). Longitudinal study on novel neuropeptides phoenixin, spexin and kisspeptin in adolescent inpatients with anorexia nervosa—Association with psychiatric symptoms. Nutr. Neurosci..

[44] Misra M., Miller K.K., Kuo K., Griffin K., Stewart V., Hunter E., Herzog D.B., Klibanski A. (2005). Secretory dynamics of ghrelin in adolescent girls with anorexia nervosa and healthy adolescents. Am. J. Physiol. Endocrinol. Metab..

[45] Sedlackova D., Kopeckova J., Papezova H., Vybiral S., Kvasnickova H., Hill M., Nedvidkova J. (2011). Changes of plasma obestatin, ghrelin and NPY in anorexia and bulimia nervosa patients before and after a high-carbohydrate breakfast. Physiol. Res..

[46] McIlwraith E.K., Loganathan N., Belsham D.D. (2018). Phoenixin Expression Is Regulated by the Fatty Acids Palmitate, Docosahexaenoic Acid and Oleate, and the Endocrine Disrupting Chemical Bisphenol A in Immortalized Hypothalamic Neurons. Front. Neurosci..

[47] Lopez-Rodriguez D., Franssen D., Sevrin E., Gerard A., Balsat C., Blacher S., Noel A., Parent A.S. (2019). Persistent vs Transient Alteration of Folliculogenesis and Estrous Cycle After Neonatal vs Adult Exposure to Bisphenol A. Endocrinology.

[48] Liu P.P., Xie Y., Meng X.Y., Kang J.S. (2019). History and progress of hypotheses and clinical trials for Alzheimer’s disease. Signal Transduct. Target. Ther..

[49] Yuruyen M., Gultekin G., Batun G.C., Yavuzer H., Akcan F.E., Doventas A., Emul M. (2017). Does plasma phoenixin level associate with cognition? Comparison between subjective memory complaint, mild cognitive impairment, and mild Alzheimer’s disease. Int. Psychogeriatr..

[50] Hofmann T., Weibert E., Ahnis A., Elbelt U., Rose M., Klapp B.F., Stengel A. (2017). Phoenixin is negatively associated with anxiety in obese men. Peptides.

[51] Neumann I.D., Landgraf R. (2012). Balance of brain oxytocin and vasopressin: Implications for anxiety, depression, and social behaviors. Trends Neurosci..

[52] Csabafi K., Jaszberenyi M., Bagosi Z., Liptak N., Telegdy G. (2013). Effects of kisspeptin-13 on the hypothalamic-pituitary-adrenal axis, thermoregulation, anxiety and locomotor activity in rats. Behav. Brain Res..

[53] Jiang J., He Z., Peng Y., Jin W., Wang Z., Han R., Chang M., Wang R. (2015). Kisspeptin-13 enhances memory and mitigates memory impairment induced by Aβ1–42 in mice novel object and object location recognition tasks. Neurobiol. Learn. Mem..

[54] Pałasz A., Janas-Kozik M., Borrow A., Arias-Carrión O., Worthington J.J. (2018). The potential role of the novel hypothalamic neuropeptides nesfatin-1, phoenixin, spexin and kisspeptin in the pathogenesis of anxiety and anorexia nervosa. Neurochem. Int..

[55] Song J., Xian D., Yang L., Xiong X., Lai R., Zhong J. (2018). Pruritus: Progress toward Pathogenesis and Treatment. BioMed Res. Int..

[56] Grover H.M., Smith P.M., Ferguson A.V. (2020). Phoenixin influences the excitability of nucleus of the solitary tract neurones, effects which are modified by environmental and glucocorticoid stress. J. Neuroendocrinol..

[57] Schalla M.A., Goebel-Stengel M., Friedrich T., Kuhne S.G., Kobelt P., Rose M., Stengel A. (2020). Restraint stress affects circulating NUCB2/nesfatin-1 and phoenixin levels in male rats. Psychoneuroendocrinology.

[58] Brown C.H. (2016). Magnocellular Neurons and Posterior Pituitary Function. Compr. Physiol..

[59] Gasparini S., Stein L.M., Loewen S.P., Haddock C.J., Soo J., Ferguson A.V., Kolar G.R., Yosten G.L.C., Samson W.K. (2018). Novel regulator of vasopressin secretion: Phoenixin. Am. J. Physiol. Regul. Integr. Comp. Physiol..

[60] Könczöl K., Pintér O., Ferenczi S., Varga J., Kovács K., Palkovits M., Zelena D., Toth Z. (2012). Nesfatin-1 exerts long-term effect on food intake and body temperature. Int. J. Obes..

[61] Muir L.A., Neeley C.K., Meyer K.A., Baker N.A., Brosius A.M., Washabaugh A.R., Varban O.A., Finks J.F., Zamarron B.F., Flesher C.G. (2016). Adipose tissue fibrosis, hypertrophy, and hyperplasia: Correlations with diabetes in human obesity. Obesity.

[62] Ojha A., Ojha U., Mohammed R., Chandrashekar A., Ojha H. (2019). Current perspective on the role of insulin and glucagon in the pathogenesis and treatment of type 2 diabetes mellitus. Clin. Pharmacol..

[63] Del Prato S., Marchetti P. (2004). Beta- and alpha-cell dysfunction in type 2 diabetes. Horm. Metab. Res..

[64] Oram R.A., Sims E.K., Evans-Molina C. (2019). Beta cells in type 1 diabetes: Mass and function; sleeping or dead?. Diabetologia.

[65] Yosten G.L.C. (2018). Alpha cell dysfunction in type 1 diabetes. Peptides.

[66] Yang Y., Lv Y., Liu J., Zhang S., Li Y., Shi Y. (2020). Phoenixin 20 promotes neuronal mitochondrial biogenesis via CREB-PGC-1alpha pathway. J. Mol. Histol..

[67] Wang J., Zheng B., Yang S., Tang X., Wang J., Wei D. (2020). The protective effects of phoenixin-14 against lipopolysaccharide-induced inflammation and inflammasome activation in astrocytes. Inflamm. Res..

[68] Zeng X., Li Y., Ma S., Tang Y., Li H. (2020). Phoenixin-20 Ameliorates Lipopolysaccharide-Induced Activation of Microglial NLRP3 Inflammasome. Neurotox. Res..

[69] Mai N., Prifti V., Kim M., Halterman M.W. (2020). Characterization of neutrophil-neuronal co-cultures to investigate mechanisms of post-ischemic immune-mediated neurotoxicity. J. Neurosci. Methods.

[70] Zhang B., Li J. (2020). Phoenixin-14 protects human brain vascular endothelial cells against oxygen-glucose deprivation/reoxygenation (OGD/R)-induced inflammation and permeability. Arch. Biochem. Biophys..

[71] Singh V., Roth S., Veltkamp R., Liesz A. (2016). HMGB1 as a Key Mediator of Immune Mechanisms in Ischemic Stroke. Antioxid. Redox Signal..

[72] Yang F., Huang P., Shi L., Liu F., Tang A., Xu S. (2020). Phoenixin 14 Inhibits High-Fat Diet-Induced Non-Alcoholic Fatty Liver Disease in Experimental Mice. Drug Des. Dev. Ther..

[73] Sun G., Ren Q., Bai L., Zhang L. (2020). Phoenixin-20 suppresses lipopolysaccharide-induced inflammation in dental pulp cells. Chem. Biol. Interact..

